# Supplementary material for: Using Acanthamoeba spp. as a cell model to evaluate Leishmania infections
Source: PLoS Negl Trop Dis. 2024 Oct 2;18(10):e0012517. doi: 10.1371/journal.pntd.0012517 (PMC11472918; doi:10.1371/journal.pntd.0012517)
Supplement: Attachment — Submitted filename: Response to Reviewers.docx [file pntd.0012517.s002.docx]

We are grateful to the reviewer for taking the time to carefully review the manuscript and give detailed and constructive comments, which has helped to improve this paper. Below is our point-by-point response to each respective comment.

Reviewer #1: Although Acanthamoeba have been isolated from the gut of wild Aedes aegypti mosquitoes (Otta at al, 2012) and the same species has been found to carry Leishmania spp. In Brazil (Coelho et al, 2017). It is not clear that Leishmania promastigotes are ever likely to encounter Acanthamoeba often.  However, the present study aims to investigate the possible use of Acanthamoeba as a model system to study Leishmania promastigotes interaction with phagocytes. It is possible that by comparing the interaction of Leishmania promastigotes with Acanthamoeba and vertebrate derived macrophages important information may be obtained. Indeed, this has been fruitful in investigating the interactions of both Acanthamoeba and macrophages with Legionella bacteria (as the authors allude to). Perhaps the use of *Dictyostelium discoideum* would be even more productive given this organism’s famous genetic tractability and the large experimental tools available in this system?

I agree with the reviewer; heterologous non-mammalian hosts may highlight the similarities and differences between different hosts in Leishmania pathogenesis, and they complement studies in mammalian systems and those using other genetic approaches. The *D. discoideum* is an amoeba used as a model host because it is haploid, has a sequenced genome, and mutants are available. It has been employed as a general model to aid our understanding of phagocytic processes, autophagy‐related pathologies, and others. However, there is one major drawback: this ameba can be grown optimally at 22° C, but their growth is significantly inhibited above 25°C and cannot survive at temperatures exceeding 27°C. In contrast, *Acanthamoeba* is associated with various human pathogens. *Acanthamoeba* spp has become a versatile and robust model for analyzing host-pathogen interactions in the last few years.

Our findings show that the acanthamoeba-pathogen interaction goes much further than what is seen with *Legionella pnemophila*. The results showed us that the intracellular growth of *Leishmania* spp within Acanthamoeba is like the *Leishmania* infection process in macrophages. However, further analyses are needed to scrutinize the biological processes and pathways and their association with known parasitic invasion and evasion mechanisms.

*Acanthamoeba* are attractive because they can be maintained at different temperatures, and they are economical, ethically expedient (adequate surrogate models), and facile handle for exploratory research of *Acanthamoeba -Leishmania* interactions. Beyond that, this model can potentially be screened for new antileishmanial drugs.

Figure 2. It is unusual for each of the growth points to be labelled with the number of trophozoites? This makes the figure quite cluttered, and it would be much more helpful to include error bars here.

We didn’t see it that way. Thanks for pointing out this inconsistency. Figure 2 was changed as recommended by the reviewer.

Line 348. This might be clearer as “Macrophages represent the major effector cells that phagocytose parasites within vertebrate hosts”.

This is an excellent suggestion. We added “within vertebrate hosts,” as suggested by the reviewer.

Line 397. It may not be accurate to describe the mannose binding proteins identified by de Souza Gonçalves et al, 2019 as being members of the ConA-like superfamily as there is only a 53 amino-acid sequence that is modelled to fold in a similar way to ConA? The word “ConA” should be spelled out in full as Concanavalin A anyway.

Thank you for bringing this to my attention. The paragraph has been rewritten.

A. castellanii can also engulf fungi that are potential pathogens, such as Cryptococcus neoformans, Histoplasma capsulatum, Blastomyces dermatitides, Fusarium solani, and Sporothrix schenckii [48]. Mannose-binding lectins (MBLs) play a critical role in activating antifungal responses by immune cells [49, 50]. A study has suggested that mannose-binding proteins identified in fungal recognition by both amoeboid and macrophage surfaces belong to the Conavalin A-like superfamily. Yet, the results reveal a limitation: a short sequence of 53 amino acids does not have enough resolution to predict protein folding accurately. [36]. However, data from the literature show us that macrophages and Acanthamoeba may share similar interaction mechanisms with fungal and bacteria pathogens, indicating a possible convergent evolution.

Line 251.  “* = numerous pine-like pseudopods (acanthopodia)”. “spine” not “pine” in this sentence?

Thank you for pointing out the typographic mistake. We fixed it

Line 412. Do the authors mean “remained viable and the death phase was not detected” rather than “analyzed” here?

Thank you for pointing out the typographic mistake. We fixed it

Line 416. *Italicise “L. infantum”*

Thank you for pointing out the typographic mistake. We fixed it

Line 467. There are small problems with the reference list some of which are mentioned below but in addition there is in consistency in the use of italics for species names.

Thank you for pointing out the reference mistake. The reference section was all reviewed.

Line 497. Reference 9 is not formatted correctly, and some words run into each other.

Thank you for pointing out the reference mistake. The reference section was all reviewed.

Line 550. References 23 and 25 are the same paper.

Thank you for pointing out the reference mistake. The reference section was all reviewed.

Line 521. Missing “B” in  author Bajgar name

Thank you for pointing out the typographic mistake. We fixed it

Line 574. Reference 29 is not formatted correctly.

Thank you for pointing out the typographic mistake. We fixed it

Line 670. Reference 52 is not formatted correctly.

Thank you for pointing out the typographic mistake. We fixed it

Line 490 “quest for new drugs” not “que for new drugs” in the title of the Hefnawy paper.

Thank you for pointing out the typographic mistake. We fixed it

Reviewer #2: the presented data are not clear and not conclusive, why using an amoebae instead of a macrophage?

*Leishmania*-macrophage interactions still need to be totally known, and so, we do not entirely understand the infection dynamics and parasite persistence.

The innate immunity pathways are widely conserved in nature; *Acanthamoeba* resembles human macrophages in many ways, particularly in their cell surface receptors, interactions, phagocytic activity, and phagolysosome processing. *Acanthamoeba-Leishmania* interactions may highlight the similarities and differences in Leishmania pathogenesis, and this model interaction may complement information on the interaction of infective promastigotes with macrophages and other phagocytes obtained since the 70s. Beyond, *Acanthamoeba* spp have evolutionarily conserved immunity and offer the opportunity to understand with a particular focus on innate immune-related against *Leishmania* of clinical importance. However, *Acanthamoeba* do not express cytokine receptors and do not produce chemokines, differently which is observed with macrophages in initiating innate and acquired immune response activation cascades during leishmania infections. The fact that *Acanthamoeba* lacks an innate and adaptive immune system in the same way as present in vertebrates simplifies the dissection of part of innate immunity during *Acanthamoeba-Leishmania* interactions. The first steps in interaction macrophage-*Leishmania* are critical in determining the evolution of the disease.

The present study aims to assess the *Acanthamoeba*, a professional phagocyte non-eukaryotic, as a model system to study *Leishmania* interaction. Our findings showed the viability and intracellular growth of *Leishmania* spp within *Acanthamoeba*, with similarity in the *Leishmania* infection process in macrophages. The comparison of results of *Leishmania* promastigotes with Acanthamoeba with vertebrate-derived macrophage interactions may obtain relevant information. Indeed, this has been fruitful in investigating the interactions of both Acanthamoeba and macrophages with *Legionella* bacteria that utilize the same genes to grow intracellularly in both human macrophages and amoebae. Most importantly, heterologous organisms such as *Acanthamoeba* have unique advantages: economical, ethically expedient, and facile for exploratory research of *Acanthamoeba -Leishmania* interactions.
