# Supplementary material for: Using Acanthamoeba spp. as a cell model to evaluate Leishmania infections
Source: PLoS Negl Trop Dis. 2024 Oct 2;18(10):e0012517. doi: 10.1371/journal.pntd.0012517 (PMC11472918; doi:10.1371/journal.pntd.0012517)
Supplement: Attachment — Submitted filename: response Reviewer_julho 2024.docx [file pntd.0012517.s003.docx]

Dear Editor,

We have thoroughly revised the manuscript based on Reviewer 3's comments, and the corrected file is now resubmitted.

To ensure its originality, we have placed it on the preprint web (DOI: 10.20944/preprints202407.053 4.v1) for the scientific community's global feedback. We are not just anticipating, but we are also filled with optimism about the future applications of this model, which we believe will inspire further research and innovation in the scientific community.

Our point-by-point response to the questions follows below.

Reviewer #3: The study aims to determine where Acanthamoeba is a suitable alternative model to macrophages. The reasons for this new model are not clear as the in vitro macrophages model is suitable already. However, Acanthamoeba may be more cost effective, but this is not mentioned in the manuscript. There is suggestion that Acanthamoeba

The study design could be developed further. It is limited to observation only, and there is no mechanism described. Including a macrophage control to compare and contrast behavior would be helpful. I would also suggest further characterization using confocal or electron microscopy. There is suggestion that innate immune systems are similar between Acanthamoeba and macrophages, but this has not been presented in detail. Further experimentation is needed to develop this hypothesis. I think that reference to innate immune system might only refer to the process of phagocytosis. This should be clarified. No ethical concerns, but health and safety should be observed.

Is there a reference to the use of 0.01% SDS to break open amoebae?

Yes. Thank you for pointing out the lack of this reference. We fixed it

Siddiqui R, Boghossian A, Akbar N, Khan NA. A one health approach versus Acanthamoeba castellanii, a potential host for Morganella morganii. Int Microbiol. 2022 Nov;25(4):781-788. doi: 10.1007/s10123-022-00261-w. Epub 2022 Jul 6.

We pointed out some explanations about the considerations of the Reviewer.

The study evaluated Acanthamoeba as a valuable, supplementary model to macrophages, emphasizing its unique advantages over other models rather than proposing it as a replacement.

There are many reasons for the use of this model.

1)     Macrophages from various sources have been used to study host-parasite interaction. These include bone marrow-derived murine macrophages, peritoneal murine macrophages, human peripheral blood monocyte-derived macrophages, and immortalized monocyte-like cell lines such as THP-1 cells. Each of these has some disadvantages. For instance, the primary macrophages are incompatible with high-throughput screening as their isolation is time-consuming, the yields are low, and there is a significant variation between batches. On the other hand, the malignant origin of the monocyte-like cell lines, such as THP-1, makes them a less physiologically accurate model for investigating host-parasite interaction. These limitations underscore the urgent need for an alternative model like Acanthamoeba.

2)      The use of Acanthamoeba as a model for the interaction of Leishmania and drug screening is not only scientifically sound but also free from ethical concerns. It aligns with the principles of the 3Rs, replacing the use of animals, and underscores its potential for ethical research.

3)     There are many gaps in information relevant to how *Leishmania* subverts macrophage functions

The study design, based on Light microscopical examination and Fluorescence microscopy images of Acanthamoeba–Leishmania co-culture, provides valuable insights. It shows that Leishmania is in vacuoles and can subvert the microbicidal activity and replicate inside Acanthamoeba spp. However, it's important to be aware that the mechanisms or strategies for manipulating activities, including receptors, nitric oxide and oxygen radical generation, cytokine production, engulfment, and phagolysosome processing, are not the goals of this study.

The intricate process of phagocytosis of Acanthamoeba unfolds in several fascinating phases:

1) The detection of the particle to be ingested.
2) Activation of the internalization process.
3) Formation of a specialized vacuole called a phagosome.
4) Maturation of the phagosome into a phagolysosome (This new organelle contains enzymes that can degrade the ingested particle).

Moreover, *Acanthamoeba* can distinguishing between self and non-self, a fundamental aspect of the immune system in many species.

The similarities in molecular mechanisms involved in directional motility, recognition, binding, engulfment, and phagolysosome processing between mammalian macrophages and Acanthamoeba spp. have already been demonstrated with bacteria and fungi. Our analysis shows that a significant disruption occurs when Leishmania subverts Acanthamoeba functions, enabling its replication inside them, as happens with macrophages. This reinforces the similarities in the molecular mechanisms, as demonstrated with bacteria and fungi between Acanthamoeba and mammalian macrophages. Despite this, we still need to assess the mechanism listed above. Our study only reveals that Leishmania manipulates Acanthamoeba functions, INDIRECTLY confirms the similarities in the molecular mechanisms involved in subvertion step.

The NB2 laboratory is designed to ensure the health and safety of laboratory work.  All steps of manipulation described must be carried out aseptically inside the biological laminar flow cabinet. The meticulous maintenance of sterile environments is crucial, and researchers should be fully aware of the specific procedures that should be carried out inside them to ensure health and safety in laboratory work.

  Reviewer #3: The results are limited to microscopic observations. Figure 3 is useful but hard to follow as it is not clear if Leishmania are actually inside the amoeba.
It would be useful to have subheadings in the results and it was difficult to follow with the figure legends in the middle of the text.

The authors firmly disagree with the reviewer's statement that "it is unclear if Leishmania are actually inside the amoeba." Our research provides strong evidence to the contrary, and we stand by our findings. We are confident in the accuracy and validity of our research, and we believe that our evidence is compelling.

Figures 3, 4, and 5, crucial visual aids, depict the amastigote inside Acanthamoeba vacuoles. This observation is further supported by the fact that it is akin to the microscopic view of the amastigote parasite stage in tissue specimens (macrophages), which remains the primary and most reliable diagnostic method. Moreover, figure 6 confirmed Leishmania-FIT-C inside of vacuoles.

The figure legends, which are placed in the middle of the text, serve as a guide to understanding the figures. They provide important context and details that are not immediately apparent from the figures themselves. This is a recommendation of PNDT. The reviewer can confirm into the Submission Guidelines PNTD recommends "Insert figure captions in the manuscript text, immediately following the paragraph where the figure is first cited (read order). Don't include captions as part of the figure files or submit them in a separate document.

Reviewer #3: Line 150: change parasite to amoeba

Thank you for bringing this to my attention. We fixed it
Line 159: specie should be species

Thank you for pointing out the mistake. We fixed it

Line 174: why these temperatures?

We tested two temperatures (a low and high) because of the species' promastigote temperature sensitivity. The visceral species (L. donovani and L. infantum, synonymous L. chagasi) are more temperature-resistant than the cutaneous species (L. major, L. tropica, L. mexicana, L. braziliensis, L. panamensis, and L. amazonensis).

Line 194: has 0.01%SDS been used before? please reference

Thank you for pointing out the mistake. We fixed it

Siddiqui R, Boghossian A, Akbar N, Khan NA. A one health approach versus *Acanthamoeba castellanii*, a potential host for Morganella morganii. Int Microbiol. 2022 25(4):781-788. doi:10.1007/s10123-022-00261

in result section I would suggest the use of subheadings

The authors added “ subheadings in the results section,” as suggested by the reviewer

Lines 224-227: review grammar

Thank you for pointing out the mistake. We fixed it

Line 225: Acanthamoeba move very very slowly. It would not be possible to see them move without a time lapse. Can this be explained?

Live trophozoites were active with a remarkable pleomorphism which changed from ovoid to quite elongated shapes. When moving, amoebae formed cytoplasmic projections such as wide lamellae and acanthopodia of diverse size and thickness. They move slowly by forming usually one or two pseudopodia in the direction of movement

Line 269: what is meant by 'loose vacuole'?

Thank you for bringing this to my attention. We fixed it

Lines 425-428 I don't understand the reference to the innate immune system from this sentence. There is not study of innate immune system.

Thank you for bringing this to my attention. We fixed it

I rewrote the sentence: In the present study, Leishmania spp. can subvert the functions of A. castellanii and A. polyphaga, similar to what happens during the innate immune response of macrophage mammals.

Point of my view

The similarities molecular mechanisms involved in directional motility, recognition, binding, engulfment, and phagolysosome processing between mammalian macrophages and Acanthamoeba spp., have already demonstrated with bacteria and fungi.

Both Acanthamoeba and macrophages uptake microbes via phagocytosis. This actin-dependent process involves monomeric G-actin polymerizing into filamentous F-actin, resulting in pseudopod formation and cell motility. To this end, much of our understanding of the structure and function of the actin, myosin, profiling, and actin-binding proteins come from Acanthamoeba, and it has long been studied as a model eukaryotic cell with emphasis on the actin cytoskeleton. At the cellular level, two convertible states of cytoplasm

are observed in Acanthamoeba and macrophages, i.e., endoplasm and ectoplasm. Endoplasm (seen at the cell center, hence the name) is fluid, while ectoplasm under the cell membrane is gelled and comparatively static. During active locomotion, the endoplasm flows forward. As the fluid endoplasm reaches the ‘‘hyaloplasm’’, a transparent form of ectoplasm, the flow is diverted towards the membrane; the endoplasm is gelled

to form ectoplasm. These transformations are visible in human macrophages and Acanthamoeba and exhibit remarkable similarity at the molecular level in structure, function, and biochemical regulation of phagocytosis and cellular motility.

Post-uptake, both Acanthamoeba, and macrophages demonstrate several similarities in oxidative metabolism. For example, both exhibit cyanide-insensitive O2 uptake and increased O2 consumption during phagocytosis by expressing a respiratory burst-like oxidase system responsible for stimulated O2 uptake during phagocytosis, leading to O2 formation. Thus, phagocytosis in Acanthamoeba exhibits many similarities to the macrophages, including increased cyanide-insensitive O2 uptake during phagocytosis, stoichiometric conversion of O2 to oxidants to kill ingested bacteria, exhibiting a direct relationship between the number of phagocytic vesicles produced per cell and the extent of oxidant production and the presence of a b-type cytochrome in phagolysosomal membranes suggesting that the enzyme complexes responsible for the respiratory burst in both Acanthamoeba and macrophages are analogous which highlights a possible evolutionary relationship ). Acanthamoeba has a superoxide-generating NADPH oxidase to produce bactericidal superoxide, similar to macrophages, which use an NADPH oxidase. Overall, much of our understanding of the cytoskeleton, cell motility, engulfment using pseudopodia, invagination, phagolysosome formation, re-cycling of the membrane, and the underlying mechanisms comes from studies on Acanthamoeba. The similarities of these processes at the morphological, molecular, and biochemical details and the functional level are outstanding.

Given some dissimilarities, it may seem far-fetched to propose that Acanthamoeba may have had an evolutionary relationship with macrophages, but yes, a parallel evolution.

Reviewer #3: The study is novel, but I am not sure of the relevance to the 'real world' and as a model for Leishmania-macrophage interactions, seeing as we already have a good model in vitro.

I can see that this study provides a good foundation to understanding microbial interactions. Seeing as there are other amoebae that harbor kinetoplastid endosymbionts, this is a good foundation to understanding how this relationship has developed. Therefore, I would ask if PNTD is the most appropriate journal for this study.

This unique perspective challenges the traditional view and opens up new avenues for research. Despite its well-established status, the macrophage model's limitations are starkly evident in the failure of all research with macrophages to uncover how Leishmania subverts macrophage functions. This underscores the urgent need for a new, more effective model, as the current one is clearly not revealing essential information about subvention of leishmania.

This in vitro model has the potential to be a game-changer in our understanding of the molecular mechanisms underlying host-parasite cellular interactions. It could reveal how Leishmania can molecularly manipulate the host into a peaceful coexistence or destruction. What are the conditions for either strategy? This model could also pave the way for studying the genomics and proteomics of parasite virulence, particularly in the context of host molecular signaling pathways that generate nitric oxide (NO), tumor necrosis factor alpha (TNF-alpha), Interleukin-12 (IL-12), Radical Oxygen Species (ROS), among others. The potential benefits of this model are vast and exciting, offering a wealth of new research opportunities.

This paper presents a groundbreaking study that significantly advances our understanding of Leishmania's behavior. PLOS Neglected Tropical Diseases publishes original research articles that are essential to the NTD and wider health communities.

Reviewer #3: The conclusions are only partly supported by the data. Acanthamoeba can host Leishmania but there is not sufficient understanding of the molecular aspects of the Leishmania–Acanthamoeba interaction considering innate immunity. the public health relevance is not elaborated and due to this I am not sure if this is a relevant journal for this study?

This model has many advantages. Firstly, it reproduces what happens with macrophages. Secondly, it enables the generation of larger, more uniform cell batches for screening campaigns. Its defining characteristics are the model's simplicity, ease of use, and cost-effectiveness. However, its most significant advantage is its speed, bringing efficiency to your work. Lastly, this approach, in line with the 3Rs principles, replaces the need for animal use in the cultivation of Leishmania and drug screening.

PLOS Neglected Tropical Diseases publishes original research articles that are essential to the NTD and wider health communities.  The journal considers any papers that examine the NTDs

For instance:

1) Amissah NA, Gryseels S, Tobias NJ, Ravadgar B, Suzuki M, Vandelannoote K, Durnez L, Leirs H, Stinear TP, Portaels F, Ablordey A, Eddyani M. Investigating the role of free-living amoebae as a reservoir for Mycobacterium ulcerans. PLoS Negl Trop Dis. 2014 Sep 4;8(9):e3148. doi: 10.1371/journal.pntd.0003148.
2) Nana Ama Amissah; Sophie Gryseels,  Nicholas J. Tobias, Bahram Ravadgar, Mitsuko Suzuki, Koen Vandelannoote, Lies Durnez, Herwig Leirs, Timothy P. Stinear, Françoise Portaels, Anthony Ablordey and Miriam Eddyani. Investigating the Role of Free-living Amoebae as a Reservoir for Mycobacterium ulcerans. DOI: [10.1371/journal.pntd.0003148](https://doi.org/10.1371/journal.pntd.0003148).
3) Patrícia Albuquerque, André Moraes Nicola, Diogo Almeida Gomes Magnabosco, Lorena da Silveira Derengowski, Luana Soares Crisóstomo, Luciano Costa Gomes Xavier, Stefânia de Oliveira Frazão, Fernanda Guilhelmelli, Marco Antônio de Oliveira, Jhones do Nascimento Dias, Fabián Andrés Hurtado, Marcus de Melo Teixeira, Allan Jefferson Guimarães, Hugo Costa Paes, Eduardo Bagagli, Maria Sueli Soares Felipe, Arturo Casadevall, Ildinete Silva-Pereira. [A hidden battle in the dirt: Soil amoebae interactions with *Paracoccidioides* spp](https://journals.plos.org/plosntds/article?id=10.1371/journal.pntd.0007742). Research Article | published 07 Oct 2019 PLOS Neglected Tropical Diseases <https://doi.org/10.1371/journal.pntd.0007742>
4) Lore Baert, Serena Rudy, Mélanie Pellisson, Thierry Doll, Romina Rocchetti, Marcel Kaiser, Pascal Mäser, Matthias Müller. [Induced pluripotent stem cell-derived human macrophages as an infection model for *Leishmania donovani*](https://journals.plos.org/plosntds/article?id=10.1371/journal.pntd.0011559). Research Article | published 02 Jan 2024 PLOS Neglected Tropical Diseases <https://doi.org/10.1371/journal.pntd.0011559>
5) Amissah NA, Gryseels S, Tobias NJ, Ravadgar B, Suzuki M, Vandelannoote K, Durnez L, Leirs H, Stinear TP, Portaels F, Ablordey A, Eddyani M. Investigating the role of free-living amoebae as a reservoir for Mycobacterium ulcerans. PLoS Negl Trop Dis. 2014 Sep 4;8(9):e3148. doi: 10.1371/journal.pntd.0003148.

Reviewer is right! Besides Paramoeba, another example of amoebae that harbor kinetoplastid endosymbionts is the genus Neoparamoeba. These amoebae are found in marine environments, and some species can be parasites of fish that cause amoebic gill disease. Similar to Paramoeba, Neoparamoeba species harbor endosymbionts called Perkinsiella amoebae. These endosymbionts are believed to be related to the kinetoplastid Ichthyobodo, a free-living flagellate. The endosymbiont's presence seems beneficial for both organisms, as research suggests a metabolic and cellular interdependence between the amoeba and the kinetoplastid.

Studying the interactions between Leishmania and Acanthamoeba for the first time holds excellent promise. This research may provide a powerful paradigm for better understanding each one's evolutionary place, sparking curiosity and offering hope for new insights and breakthroughs.
